# Factors Affecting the Perceived Social Support of Adolescent Girls and Women Aged 10–45 Years With Disabilities in Selected Sub-districts of Bangladesh

**DOI:** 10.7759/cureus.68009

**Published:** 2024-08-28

**Authors:** Munzur E Murshid, Hiromi Kawasaki, Md Moshiur Rahman, Namira Rahman Era, Md Ziaul Islam, Yoko Shimpuku

**Affiliations:** 1 Department of Health Sciences, Graduate School of Biomedical and Health Sciences, Hiroshima University, Hiroshima, JPN; 2 Maternal and Child Health, Independent General Practice, Dhaka, BGD; 3 Department of Community Medicine, National Institute of Preventive and Social Medicine, Dhaka, BGD

**Keywords:** bangladesh, adolescent girls with disabilities, women with disabilities, associated factors, perceived social support

## Abstract

Background

Perceived social support is crucial for the well-being of women and adolescent girls with disabilities. In Bangladesh, social support can significantly influence their quality of life, yet it remains understudied. This study aims to identify the factors affecting perceived social support among women and adolescent girls with disabilities in selected sub-districts of Bangladesh.

Methodology

In this cross-sectional study, a total of 152 women and adolescent girls with disabilities participated. The study was conducted in Bogura Sadar and Chapainawabganj Sadar upazilas of Bangladesh. The implementation timeline of the study was from March to April 2023. The chi-square test and multiple linear regression analysis were conducted to explore the factors affecting perceived social support among the participants in the selected sub-districts of Bangladesh.

Results

In the chi-square test, associations were noted between menarche age, male education, and household income categories with perceived social support categories. In Fisher’s exact test, an association was observed between females with partial hearing disability and perceived social support categories. The multiple linear regression analysis showed that better levels of education in men, high household income, and a younger age of menarche and partial hearing disability were the predictors of higher perceived social support among women and adolescent girls with disabilities.

Conclusions

This study underscores the multifaceted nature of perceived social support among women and adolescent girls with disabilities in Bangladesh. Interventions aimed at enhancing social support should consider identified predictors to provide tailored support for women and adolescent girls with disabilities. Enhanced social support can lead to overall well-being for this vulnerable population.

## Introduction

Perceived social support plays a crucial role in the overall well-being and quality of life of individuals, particularly for vulnerable populations such as women and adolescent girls with disabilities [1,2]. Social support encompasses emotional, informational, and practical assistance received from family, friends, and the community. It significantly impacts mental health, coping mechanisms, and the ability to navigate daily challenges [3,4]. In the context of disabilities, adequate social support can mitigate the adverse effects of societal stigma, discrimination, and marginalization [5].

In Bangladesh, women and adolescent girls with disabilities face multifaceted challenges, including limited access to education, healthcare, and employment opportunities. Cultural norms and societal attitudes often exacerbate their isolation, leading to diminished self-esteem and increased vulnerability [6]. Understanding the factors that influence their perceived social support is essential for developing targeted interventions that can enhance their well-being and integration into society.

Family dynamics play a pivotal role in shaping the perceived social support of women and adolescent girls with disabilities. Supportive family environments, characterized by strong emotional bonds and practical assistance, can enhance their sense of security and belonging [7]. On the other hand, strained family relationships or lack of understanding about disabilities can hinder the provision of adequate support.

Social support refers to “assistance with instrumental and expressive functions that comes from interpersonal relationships.” Two major influences on mental health have been identified, namely, a “direct effect” and a “buffering effect.” The former states that mental health status is directly determined by the amount of social support, while the latter states that social support functions to buffer the adverse effects on the mental health of experiencing negative life events and contracting illness [8].

For people with disabilities, the perception of social support is as important as their mental support. Identifying the factors that influence their perception of social support can help us consider ways to increase the social support they feel, which can further contribute to improving their mental health.

This study aimed to identify the factors affecting the perceived social support of women and adolescent girls with disabilities in selected sub-districts of Bangladesh. By focusing on selected sub-districts of Bangladesh, the research seeks to provide a comprehensive understanding of the local context and specific needs of this marginalized group. Understanding these factors is crucial for addressing the unique challenges faced by women and adolescent girls with disabilities and promoting an inclusive society that values and supports all its members.

## Materials and methods

In this cross-sectional study, a total of 152 women and adolescent girls with disabilities were enrolled. The study was conducted in Bogura Sadar and Chapainawabganj Sadar upazilas in Bangladesh. The study was conducted from March to April 2023. In the study, the Multi-dimensional Scale of Perceived Social Support was used. The scale has 12 items divided into friends, family, and significant other subscales. Mean scores of 1-2.9 are considered to denote low support, mean scores of 3-5 are considered to denote moderate support, and mean scores of 5.1-7 are considered to denote high support. The Bengali version of the scale was used. The Cronbach’s alpha of the entire scale was 0.868. Following the Lawshe approach [9], it was concluded that all the scale’s items scored 0.99 [10-12].

Sample size calculation

To determine the sample size, G*Power (version 3.1.9.4) was utilized [13]. A medium Cohen’s effect size of 0.13 [14], an alpha error probability of 0.05, and a power of 0.80 were considered. The analysis of variance for repeated measures within and between interactions was selected. The loss to follow-up rate was estimated at 30%. Finally, the calculated sample size was 156. Based on available resources, 152 women and adolescent girls with disabilities were recruited in the study.

Ethical approval

The review board of the National Institute of Preventive and Social Medicine, Dhaka, Bangladesh, provided approval for the study (approval number: NIPSOM/IRB/2023/07).

Participant recruitment

Two field staff were recruited for the study from two local organizations for persons with disabilities. These staff received training on receiving informed consent, interviewing participants, and collecting data. After the training, they visited the homes of women and adolescent girls with disabilities. They briefed them about the study objectives, steps, data security, and provision of privacy. Subsequently, informed consent was received from the participants. If the participant was below 18 years old, their legal guardian provided assent to participate in the study.

Inclusion criteria

We included menstruating adolescent girls with disabilities aged 10 to <18 years and women with disabilities aged 18 to 45 years old. Female peers with disabilities such as speech, visual, hearing, physical, or combined disabilities were included. Participants residing in Bogura Sadar and Chapainawabganj Sadar for more than or equal to six months were considered for inclusion. The following inclusion criteria for male peers were considered: husbands, fathers, or elder brothers of the respective female peers with disabilities aged 18-65 years and residing at the study site for more than or equal to six months.

Exclusion criteria

The following exclusion criteria were applied to female participants and their male counterparts: pregnant women, intellectually or mentally disabled individuals, those suffering from a terminal illness, and those incapable of giving informed consent or assent [15].

Data analysis

The chi-square test was conducted to explore the relationship between disability categories (physical, partial speech, partial vision, and partial hearing disability) of participants, female age, female education, household income, male age, male education categories and perceived social support categories.

Multiple linear regression analysis was conducted to explore the factors affecting perceived social support among women and adolescent girls with disabilities in the selected sub-districts of Bangladesh.

In the multiple linear regression analysis, the dependent variable was perceived social support. Independent variables included household income, male education, age at menarche, marital status, and types of disability. The selected model provided the highest R^2^ value. For the multiple linear regression, all associated variables were tested, including the selected ones. Excluded variables were female education and female age. The inclusion of excluded variables was not satisfactory considering the R^2^ value.

## Results

In this study, 15 females were in the age group of 0-17 years, 80 females were in the age group of 18-30 years, and 57 females were in the age group of 31-45 years. Their mean menarche age was 11.95 years. Female educational qualifications were divided into the following two categories: category 1 (class one to class five), and category 2 (class six to class fifteen). Category 1 educational qualification was achieved by 101 female participants, and 51 female participants acquired category 2 educational qualification. Among the women and adolescent girls with disabilities, 93 had physical disabilities, 24 had partial speech disabilities, 24 had partial visual disabilities, and 11 had partial hearing disabilities (Table 1).

**Table 1:** Demographic characteristics of females. Partial hearing disability: the individual has limited hearing ability but she can listen moderately. Partial visual disability: the individual has limited visual ability but can see moderately. Partial speech disability: the individual has speech impairment but can talk that is understandable moderately

Demographic characteristics	Number	%	Total
Age (years)			
10–17	15	9.87%	152
18–30	80	52.63%	
31–45	57	37.50%	
Education			
Class 1–5	101	66.45%	152
Class 6–15	51	33.55%	
Disability types			
Physical disability	93	61.18%	152
Partial speech	24	15.79%	
Partial visual	24	15.79%	
Partial hearing	11	7.24%	
Menarche age (years)			
9–12 years	118	77.63%	152
13–15 years	34	22.37%	

Household incomes were categorized into the following four categories: category 1 (0-6,000 BDT per month), category 2 (6,001-9,000 BDT per month), category 3 (9,001-12,000 BDT per month), and category 4 (12,001-21,000 BDT per month). Overall, 28 households were in category 1, 33 households in category 2, 60 households in category 3, and 31 households in category 4. Of the men, 49 were in the age group of 0-40 years, and 103 were in the age group of 41-65 years. Male educational qualifications were divided into the following two categories: category 1 (class zero to class five), and category 2 (class six to class sixteen). Of the male participants, 106 had achieved category 1 educational qualification, and 46 had achieved category 2 educational qualification (Table 2).

**Table 2:** Demographic characteristics of females and household incomes. BDT refers to Bangladeshi Taka (currency).

Demographic characteristics	Number	%	Total
Age (years)			
18–40	49	32.24%	152
41–65	103	67.76%	
Education			
Class 0–5	106	69.74%	152
Class 6–16	46	30.26%	
Household income (BDT)			
0–6,000	28	18.42%	152
6,001–9,000	33	21.71%	
9,001–12,000	60	39.47%	
12,001–21,000	31	20.39%	

In the chi-square test, associations were noted between females with menarche age, male education, and household income category with perceived social support categories. The respective p-values were 0.008, <0.001, and <0.001, respectively. According to Fisher’s exact test, partial hearing disability was associated with perceived social support, with a p-value of 0.001 (Table 3).

**Table 3:** Analysis of association between perceived social support categories and disability types, menarche age, female age, female education, male age, male education, and household income categories (perception of social support among females in need of support and related background). The test was applied between the perceived social support categories: (1) low, (2) moderate, and (3) high disability categories: physical, partial speech, partial vision, partial hearing disability; male age category: category 1 (0-40 years), category 2 (41-65 years); male education category: category 1 (0-5 class), category 2 (6-16 class). Female age category: category 1 (10-17 years), category 2 (18-45 years); female education category: category 1 (1-5 class), category 2 (6-15 class); household income category: category 1 (0-15,000 BDT), category 2 (15,001-21,000 BDT); menarche age: category 1 (9-12) and category 2 (13-15). There was no low perceived support response. Moderate and high perceived social support categories were compared with other variable categories. There was no association between physical, partial speech, or partial visual disability and perceived social support categories. In the chi-square test, male partner age, female peer age, and education were not associated with perceived social support categories. BDT refers to Bangladeshi Taka (currency).

Categorical variable	Category	Social support							
		Moderate			High			Total	P-value
		Observed number	Expected count	％	Observed number	Expected count	％		
Partial hearing disability	Without	68	63.1	48.2	73	77.9	51.8	141	0.001
	With	0	4.9	0.0	11	6.1	100.0	11	
Menarche age (years)	9–12	46	52.8	38.98	72	65.2	61.02	118	0.008
	13–15	22	15.2	64.71	12	18.8	35.29	34	
Male age (years)	0–40	19	21.9	38.78	30	27.1	61.22	49	0.308
	41–65	49	46.1	47.57	54	56.9	52.43	103	
Male education (class)	0–5	63	47.4	59.43	43	58.6	40.57	106	<0.001
	6–16	5	20.6	10.87	41	25.4	89.13	46	
Household income (BDT)	0–6,000	23	12.5	82.14	5	15.5	17.86	28	<0.001
	6,001–9,000	22	14.8	66.67	11	18.2	33.33	33	
	9,001–12,000	18	26.8	30.00	42	33.2	70.00	60	
	12,001–21,000	5	13.9	16.13	26	17.1	83.87	31	
Female age (years)	0–17	4	6.7	26.67	11	8.3	73.33	15	0.325
	18–30	38	35.8	47.50	42	44.2	52.50	80	
	31–45	26	25.5	45.61	31	31.5	54.39	57	
Female education (class)	1–5	47	45.2	46.53	54	55.8	53.47	101	0.53
	6–15	21	22.8	41.18	30	28.2	58.82	51	

According to the multiple linear regression analysis, the following factors affected perceived social support: partial hearing disability, menarche age, income category 1 (0-6,000 BDT), income category 2 (6,001-9,000 BDT), and males with better education (class 6-16) were significantly associated with perceived social support (ß = 0.174, p = 0.011; ß = -0.155, p = 0.027; ß = -0.365, p = <0.001; ß = -0.166, p = 0.024; and ß = 0.28, p = <0.001, respectively) (Table 4).

**Table 4:** Multiple linear regression analysis outcomes. Dependent variable: perceived social support; R^2^ = 41.1%, adjusted R^2^ = 37.4%, n = 152. Physical disability, income (9,001-12,000 BDT), and male education (class 0-5) were excluded by SPSS. BDT refers to Bangladeshi Taka (currency).

Variables	Unstandardized coefficients		Standardized coefficients	t	P-value
Independent variable	B	Standard error	Beta		
	5.94	0.403		14.725	0
Partial hearing disability	0.320	0.125	0.174	2.566	0.011
Partial speech disability	-0.014	0.045	-0.021	-0.312	0.755
Partial visual disability	-0.019	0.029	-0.043	-0.639	0.524
Age of Menarche	-0.074	0.033	-0.155	-2.239	0.027
Household income (BDT)					
0–6,000	-0.45	0.092	-0.365	-4.896	<0.001
6,001–9,000	-0.192	0.084	-0.166	-2.275	0.024
12,001–21,000	-0.025	0.089	-0.021	-0.281	0.779
Male education (class)					
6–16	0.291	0.075	0.280	3.882	<0.001

Females with partial hearing disability, younger menarche age, higher household income, and better educational levels among males were significantly associated with increased perceived social support among women and adolescent girls with disabilities. These were the significant predictors for higher perceived social support among women and adolescent girls with disabilities. The study population’s mean perceived social support score was 5.11, with a standard error of mean of 0.04 and a standard deviation of 0.48. The statistics indicate that respondents had relatively better social support.

## Discussion

In this study, women and adolescent girls with disabilities having male peers with higher education had higher perceived social support. Acquiring education creates an enlightened mind. An educated person has a greater chance to change his or her behavior if the perceived information is beneficial for themselves and the people around them [16,17]. If male peers perceive the needs and challenges of women and adolescent girls with disabilities, they will undertake initiatives to improve the condition of their female peers, particularly those requiring caregiver support. Family support, especially male peer support, is highly needed for women and adolescent girls with disabilities in a country like Bangladesh [7]. As the country has a patriarchal society [18], men decide resource allocation in the family. They should understand the challenges and needs of their female peers with disabilities.

In our study, 152 (100%) participants were dependent on their husbands or fathers. In the Social Assistance and Rehabilitation for the Physically Vulnerable (SARPV) study, 12% of the participants were involved in economic activities [19]. The study focused primarily on major cities in Bangladesh. In the major cities, relatively, there are more work opportunities [20]. This study was conducted in two sub-districts of Bangladesh, with likely fewer opportunities for income-generating activities for persons with disabilities.

Socioeconomic status emerged as a critical determinant of perceived social support. Participants from lower socioeconomic backgrounds have less access to formal support systems, such as healthcare and education, which, in turn, affects their perceived social support.

Before engaging in economic activities, women and adolescent girls with disabilities require education and skill development. Unfortunately, women and adolescent girls with disabilities face a great challenge in attaining educational qualifications due to negative perceptions in the surrounding environment, non-inclusive transport facilities, non-inclusive institutional structure, lack of training of teachers, negative attitudes from peers, financial constraints, etc. [21].

In our study, the mean educational qualification of women and adolescent girls with disabilities was class five (SD = 3.59). It was supposed that family members in this study were more supportive of the education of the girls despite the challenging economic conditions. In comparison, the report on women and adolescent girls with disabilities in Bangladesh published by the SARPV study (2008) stated that 48.6% of the participants were illiterate [22]. In a study conducted by the Center for Services and Information on Disability, due to stressful economic conditions, guardians may not be interested in the education of family members with disabilities [23].

Regarding differences in disability types, women and girls with partial hearing disability have higher perceived social support even though those with physical, partial speech, and partial visual disability also need social support. Although studies in this area are scarce, a study by Aysha et al. (2023) reported that the severity of hearing impairment did not play a significant role in their perceived social support. They conducted the study among 120 adults with hearing disability between the ages of 18 to 40 years [24]. In another study, women with physical disabilities reported facing significant barriers to mobility and access, which hindered their ability to engage with support networks. Menstruating women and adolescent girls with disabilities face numerous challenges in day-to-day life. They were five times more likely to use different bathing facilities than others in the household. Menstrual restrictions were widespread for all, but collecting water and managing menstrual materials were harder for women and adolescent girls with disabilities [25]. Women and adolescent girls with disabilities need customized support networks based on their type and severity of disability.

Strengths and limitations of the study

This is the first study to identify associated factors of perceived social support among women and adolescent girls with disabilities in Bangladesh. This evidence will help policymakers and administrators in developing interventions or policies to improve perceived social support among women and adolescent girls with disabilities. As the study focused on specific sub-districts of Bangladesh, it depicted concrete evidence in the local context.

At the same time, the sample size and geographic focus on selected sub-districts may limit the generalizability of the findings. Future research may aim to include a broader demographic to validate these findings across different regions of Bangladesh. Additionally, longitudinal studies could provide insights into how perceived social support evolves for women and adolescent girls with disabilities. There was a chance of social desirability bias. The research team ensured a congenial environment to avoid bias.

## Conclusions

This study revealed several key findings. Based on the chi-square test, perceived social support categories were linked to females with a partial hearing disability, menarche age, household income, and male education category. According to the multiple linear regression analysis, partial hearing disability, younger menarche age, higher household income, and better male education levels were significant predictors of perceived social support for women and adolescent girls with disabilities. Understanding the dynamics is crucial for developing targeted interventions that address the unique challenges faced by women and adolescent girls with disabilities. The results of this study can be used to determine the priority of the subjects by focusing on the disability status, menarche age of females, household income, and educational background of the men to be supported.
